# Bioactivation of the β-Amyloid Precursor Protein-Cleaving Enzyme 1 Inhibitor Atabecestat Leads to Protein Adduct Formation on Glutathione S-Transferase Pi

**DOI:** 10.1021/acs.chemrestox.5c00070

**Published:** 2025-05-06

**Authors:** Megan Ford, Paul J. Thomson, Adam Lister, Jan Snoeys, Laurent Leclercq, Filip Cuyckens, Dean J. Naisbitt, Xiaoli Meng

**Affiliations:** ‡ Department Pharmacology and Therapeutics, University of Liverpool, Liverpool L693GE, U.K.; † Translational Pharmacokinetics Pharmacodynamics and Investigative Toxicology, Johnson & Johnson, 2340 Beerse, Belgium

## Abstract

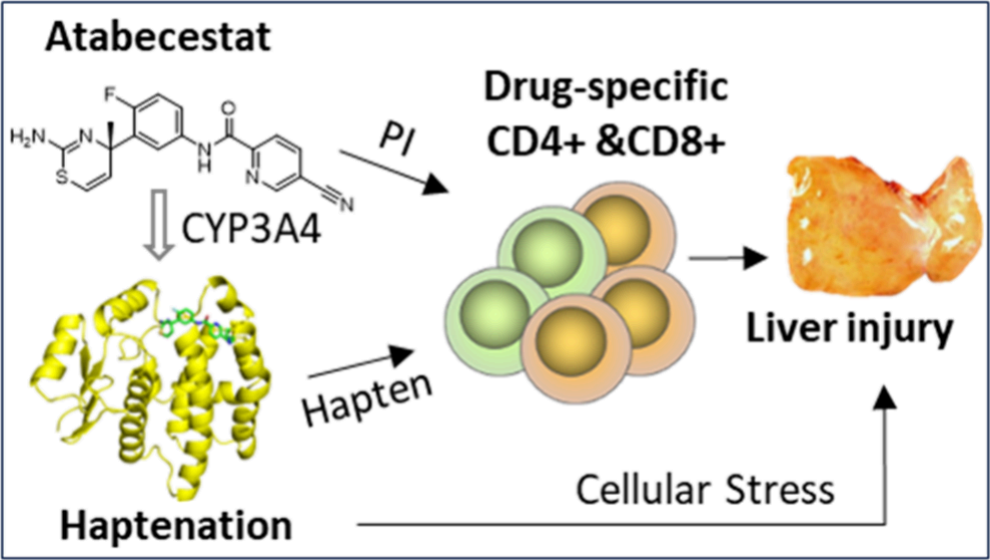

Exposure to atabecestat is associated with liver injury, which subsequently led to its withdrawal from development. Previous studies of patients with atabecestat induced liver injury identified T cells responsive to atabecestat and its metabolites, indicating that immune-mediated mechanisms are involved. As irreversible protein modification is suspected to drive immunogenicity, this study aimed to characterize potential atabecestat protein adducts using HSA, GSTA1, and GSTP as model proteins. We have shown that atabecestat only formed a cysteine adduct on GSTP in the presence of metabolic systems, highlighting the important role of bioactivation in adduct formation and selectivity for the binding interaction.

Atabecestat (ABCT), a β-Amyloid Precursor Protein-Cleaving Enzyme 1 (BACE 1) inhibitor, was discovered in Shionogi & Co., Ltd. and developed by Janssen Research & Development LLC and Shionogi & Co., Ltd. for the treatment of Alzheimer’s disease. Unfortunately, the development of ABCT was discontinued due to significant elevation of liver enzymes^1^ and later, along with other BACE inhibitors, dose-related declines in cognition. What caused the ABCT liver injury remains unknown. However, a GWAS study revealed that ALT elevations might be linked to innate immune activation pathways.^2^ Furthermore, histological examination of inflamed liver from a patient exposed to ABCT with serious liver injury revealed an infiltration of cytotoxic T lymphocytes, suggesting an immune pathogenesis.^3^ CD4+ T cells responsive to ABCT and its stable metabolites, diamino thiazine (DIAT) and N-acetyl DIAT, were also detected in peripheral blood from patients with liver injury, indicating ABCT and its stable metabolites can activate T cells through direct interaction with immune receptors.^4^ However, bioactivation studies showed high levels of metabolism-dependent covalent binding to plasma proteins in rats administrated with ^14^C-ABCT. When incubated with human hepatocytes, a cross-linking adduct resulting from the addition of ABCT to GSH and Lys120 on GSTA1 was also detected. However, the mechanisms of protein adduct formation remain to be defined.^5^ Therefore, this study aimed to explore the potential chemical mechanisms of ABCT covalent binding to proteins using glutathione S-transferase Pi (GSTP), glutathione S-transferase alpha (GSTA), and human serum albumin (HSA) as model proteins.

We first looked at the reactions between ABCT and a model nucleophile, glutathione (GSH). ABCT was incubated with GSH with or without a metabolic system consisting of CYP3A4, which was previously shown to play a role in the activation of ABCT^5^ or human liver microsome. The resulting adducts were analyzed by LC-MS. ABCT contains a nitrile moiety, which can form a reversible thioimidate ester with thiols.^6^ When ABCT (1 mM) was directly incubated with GSH (1 mM), at 37 °C for 16 h, the expected adduct with full GSH molecular mass (*m*/*z* 675) was not detected; instead, an adduct with a protonated molecular ion at *m*/*z* 529 was observed. It has been reported the initial thioimidate ester formed between the nitrile and GSH is not stable and can undergo further rearrangement to form the cyclic thiazoline structure through the reaction between the imine nitrogen and the α-carbon of the cysteine^6^ (Scheme 1, GSH adduct I). Fragment ions derived from the precursor ion at *m*/*z* 529 include *m*/*z* 453, *m*/*z* 350, *m*/*z* 264, and *m*/*z* 162, which are consistent with the proposed cyclic thiazoline structure (Figure S2A). Interestingly, when ABCT was incubated with GSH in the presence of CYP3A4 or HepG2 cell lines (seeded at 1 × 10^6^ in 6-well Nunc plates overnight at 37 °C and 5% CO_2_), the same adduct was also detected, albeit at higher levels in the presence of CYP3A4 (Figure S3).

**Scheme 1:**
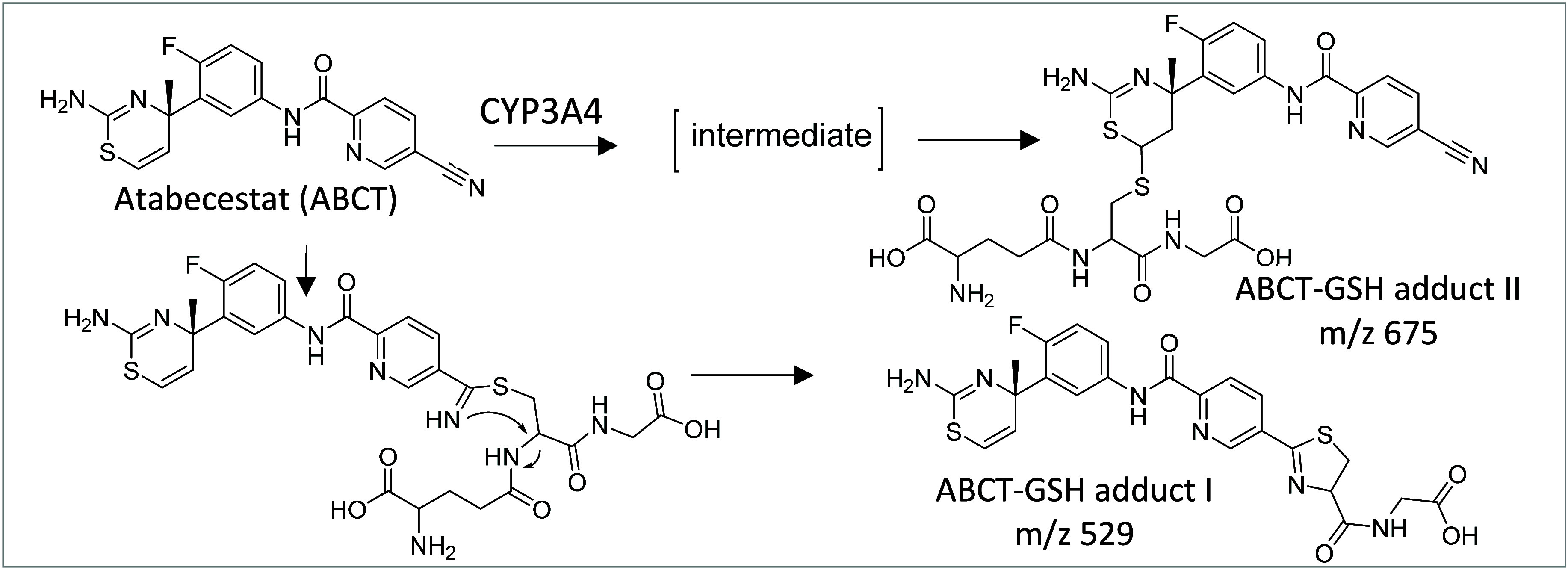
Atabecestat Glutathione Conjugates Are Formed through Either Direct Binding or Reactive Intermediates Formed by CYP3A4-Mediated Bioactivation

On the other hand, when incubated with GSH (0.2–0.4 mM) in the presence of CYP3A4 (100pmol/mL) for two h at 37 °C, an adduct at *m*/*z* 675 was detected, indicating the full GSH molecule was attached to ABCT (Scheme 1, GSH adduct II). Characteristic fragment ions including *m*/*z* 292 and *m*/*z* 368 derived from ABCT and other fragment ions derived from the adduct such as *m*/*z* 546, *m*/*z* 470, and *m*/*z* 443 indicate addition of GSH on C6 or C5 of thiazine (Figure S2B). The C6 location was confirmed by H1 NMR studies. The pathways leading to the addition of thiazine are intriguing. Multiple potential reactive intermediates including an epoxide formed on C5=C6, a radical between C5 and C6, a sulfoxide, and thiazinium tautomers are proposed. However, no experimental data could provide confirmative evidence.^5^ Interestingly, coincubation of ABCT with CYP3A4 and cofactors in the presence of GSTA led to an increase in the formation of ABCT-GSH adducts at C6, whereas coincubation with GST Mu and GSTP resulted in a reduction of the adduct, indicating the formation of ABCT-GSH adducts at C6 requires a prior metabolic activation as well as catalysis by GSTA1.^5^ We speculate that the ABCT reactive intermediates generated by CYP3A4-mediated bioactivation could form a labile adduct with GSTA1 that is oriented in the proximity of the GSH binding site, facilitating GSH conjugation. As demonstrated in the computational modeling, binding of ABCT to Lys120 placed ABCT in the correct orientation for further reaction with GSH (Figure 1). However, this may not be the same case for the addition of ABCT to GSTMu and GSTP, where binding to cysteine residues on proteins could lead to a reduction in GSH conjugation.

**Figure 1:**
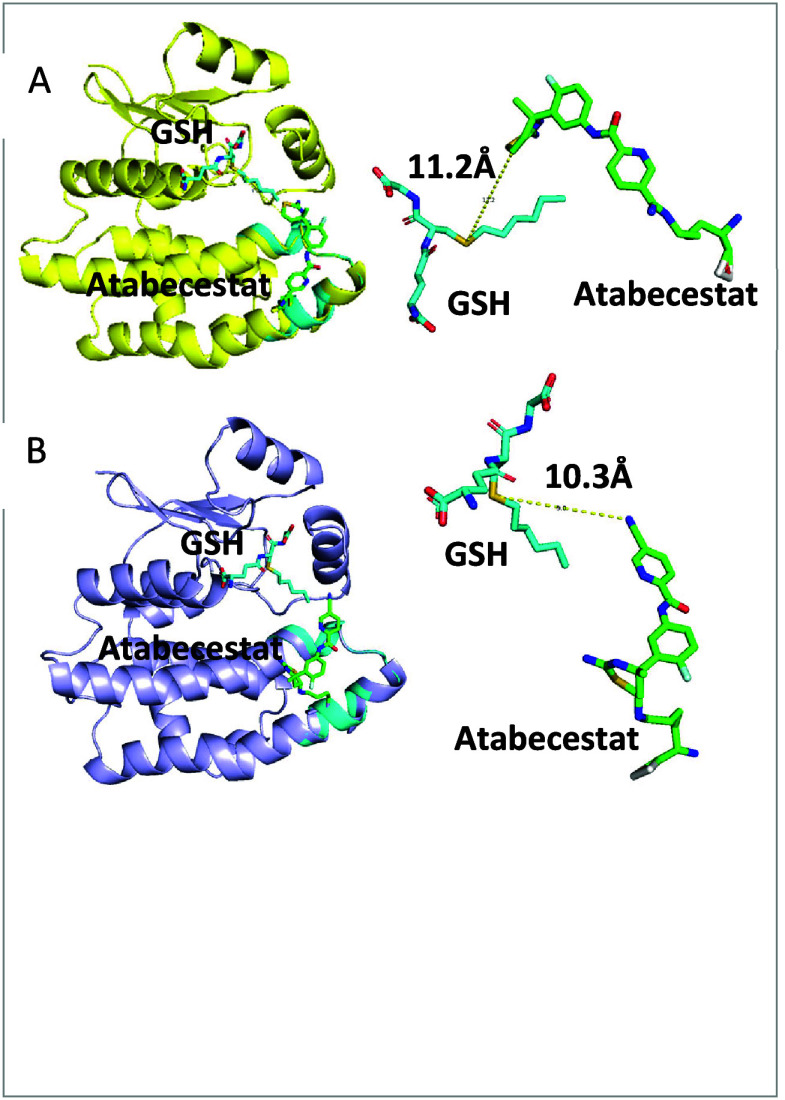
Computational modeling shows binding of ABCT to Lys120 facilitates adduction to GSH. Covalent binding of ABCT (green) to Lys120 on GSTA (PDB code 1K3L) either through the nitrile group (A) or the C6 of the thiazine (B) places ABCT in close contact with GSH (cyans), facilitating cross-linking adducts formation. Images are illustrated by PyMOL (The PyMOL Molecular Graphics System, Version 1.3 Schrödinger, LLC.).

Covalent binding of ABCT to other off-target proteins was further investigated using model proteins such as GSTP and HSA that are known targets for numerous electrophilic drugs and drug metabolites.^7^ The GSTP protein was generated by transfecting a histidine tagged human GSTP gene into BL21 cells. GSTP was isolated from cell lysate by using HIS-Select Nickel Affinity Gel followed by the Bradford protein quantification assay before incubation with ABCT. Purified GSTP captured on nickel beads (40 μM), GSTA1 (0.5 μg/μL), and HSA (6.6 mg/mL) was incubated with ABCT (0.1 mM) in the presence of metabolic systems consisting of either human recombinant CYP3A4 (100 pmol/mL) or human liver microsomes (1 mg/mL, prepared in house) with NADPH. Incubations with a range of glutathione concentrations were also included to elucidate the impact of GSH on protein adduct formation. After 16 h of incubation, HSA and GSTA were separated from unreacted ABCT or its metabolites by SDS-PAGE (Figure S4), followed by in gel digestion using previous protocols.^8^ GSTP captured on nickel beads were purified and digested (Supplementary methods). The resulting tryptic peptides were further cleaned using C18 ZipTips for LC-MS/MS analysis. Samples were analyzed using a Triple TOF 6600 mass spectrometer (Sciex) coupled to an Eksigent NanoLC Ultra HPLC system. MS was operated as described in previous methods.^9^

LC-MS/MS data were searched against the reviewed human proteome (UniProt/SwissProt accessed October 2018), using ProteinPilot software v5.0, incorporating ABCT modification of lysine and cysteine (+367.1). Manual annotation of MS/MS spectra was also performed to confirm adduct formation. Despite an extensive search using both software and manual de novo sequencing, we failed to identify any ABCT modified peptides derived from HSA or GSTA. This could be due to the stability of adducts formed on these proteins. An ABCT-plasma complex formed in plasma from rats dosed with ^14^C-ABCT was completely undetectable after sample preparation under basic conditions, indicating that the complex may contain labile adducts (Figure S5). This contradicts the C6 GSTA1 adduct detected inthe previous study.^5^ These labile adducts may be formed on proteins through a thiazinium intermediate or sulfoxide as suggested by Leclercq et al.^5^ The stability of ABCT adducts formed on different proteins varies, making it difficult to detect these adducts. In contrast, a low abundant ion corresponding to a ABCT-modified GSTP peptide^45^ASC[ABCT] LYGQLPK^54^ was detected. Figure 2 shows a representative MS/MS spectrum for a triply charged ion of *m*/*z* 482.885, which corresponds to the tryptic peptide ^45^ASCLYGQLPK^54^ with a mass addition of 367.1174 Da, indicating the presence of ABCT. The presence of the characteristic fragment ion derived from ABCT (*m*/*z* 292.0923) during collision induced dissociation provided further evidence of the modification. The modification site (Cys47) was confirmed by the presence of ABCT-modified b3 ion (*m*/*z* 629.1777), b4 ion (*m*/*z* 742.2621), and y9 ion (*m*/*z* 1090.4189), which all show a mass addition of 367 Da. This adduct was not detected in the incubation without metabolic systems, indicating it may be formed on the C6 of thiazine. Unfortunately, the absolute structure of the adduct could not be determined in the current study. Nonetheless, various factors may potentially contribute to the observed low abundance of ABCT modification: the low levels of reactive intermediates generated by metabolic systems, the high reactivity of these intermediates hindering their escape from the CYP active site, and the reversible nature of the adduct.

**Figure 2:**
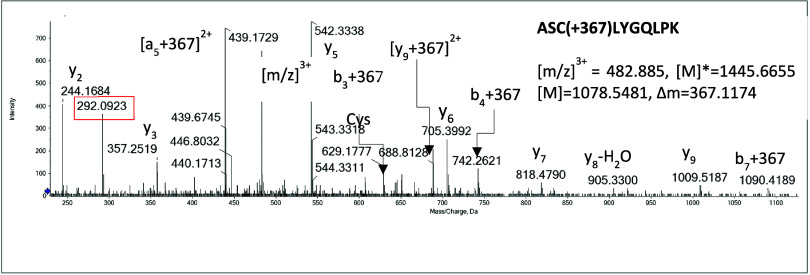
Mass spectrometric characterization of ABCT-GSTP adducts. A representative MS/MS spectrum of a triply charged ion at *m*/*z* 482.885 corresponds to the GSTP peptide ^45^ASCLYGQLPK^54^ with ABCT modification at Cys47.

Previous studies have shown that ABCT and its stable metabolites can activate T cells through direct interaction with the immune system, which may contribute to ABCT-induced liver injury.^4,10^ In this study, our data show that ABCT can form protein adducts with cysteine residues on GSTP *in vitro*. The detection of these adducts only in the presence of metabolism highlights the important role of bioactivation. The haptenation of hepatocellular proteins by ABCT reactive metabolites could either trigger cellular stress or activate hapten-specific T cells. However, further work is required to identify how these haptens interact with the immune system and their contribution to ABCT-induced liver injury.

## Abbreviations

ABCT: Atabecestat
DIAT: diamino thiazine
SDS-PAGE: SDS-polyacrylamide gel electrophoresis
GSTP & GSTA: glutathione transferase Pi & alpha
HSA: and human serum albumin.
