# Supplementary material for: Bioactivation of the β-Amyloid Precursor Protein-Cleaving Enzyme 1 Inhibitor Atabecestat Leads to Protein Adduct Formation on Glutathione S-Transferase Pi
Source: Chem Res Toxicol. 2025 May 6;38(5):812–5. doi: 10.1021/acs.chemrestox.5c00070 (PMC12093359; doi:10.1021/acs.chemrestox.5c00070)
Supplement: Supplementary file 1 — tx5c00070_si_001.pdf [file tx5c00070_si_001.pdf]

Supporting Information

Supporting Information

Bioactivation of the β-Amyloid Precursor Protein-Cleaving Enzyme 1 inhibitor atabecestat leads to protein adduct formation on glutathione S-transferase Pi

Megan Ford[‡], Paul J. Thomson[‡], Adam Lister[‡], Jan Snoeys[†], Laurent Leclercq[†], Filip Cuyckens[†], Dean J. Naisbitt[‡], Xiaoli Meng[‡]*

[‡]Department Pharmacology and Therapeutics, University of Liverpool, L693GE, UK; [†] Translational Pharmacokinetics Pharmacodynamics and Investigative Toxicology, Johnson & Johnson, Beerse, Belgium

*Corresponding Author

Dr Xiaoli Meng

Department of Pharmacology, University of Liverpool, Sherrington Building, Ashton Street, Liverpool L69 3GE, England

Telephone: 0044 151 7956066; e-mail: xlmeng@liverpool.ac.uk

Table of Contents

Supplementary methods **1**

Supplementary Figures **6**

Figure S1 6

Figure S2 7

Figure S3 8

Figure S4 9

Figure S5 10

References 11

Supplementary methods

Cell culture. HepG2 cells were previously transfected with 3xflag tag CYP3A4 maintained in F1 media supplemented with puromycin (0.5µg/mL) to select for cells expressing CYP3A4. Expression was verified by western blotting and microscopy (fluorescence) prior to use. HepG2 control cell line (without transfection) was cultured in F1 media alone. F1 medium was made by supplementing RPMI 1640 (Sigma Aldrich, St. Louis, MO, USA) with FBS (foetal bovine serum) (10%; Invitrogen, Waltham, MA, USA), L-glutamine (2mM; Sigma Aldrich, St. Louis, MO, USA), HEPES (25mM; Sigma Aldrich, St. Louis, MO, USA) and penicillin-streptomycin solution (penicillin 100IU/ml; streptomycin 100µg/ ml; Sigma Aldrich, St. Louis, MO, USA).

HepG2 viability curve with atabecestat was performed before proteomics dosing experiments were performed. HepG2 cells were seeded 20,000 cells/well on 96 flat bottom cell culture plate (100µL F1 media). Cells were incubated overnight 37 degrees. Cells were treated in triplicate with an additional 100 µL of F1 media containing 2x concentration of atabecestat or 100uL F1 media for control wells. Cells were incubated with 10uM-2mM atabecestat for 48hrs. Cell Titer-Blue® Cell Viability Assay (Promega) was then carried out following the manufacturer's instructions. A viability curve was then created by converting average absorbance to % viability of the control wells.

Atabecestat-GSH conjugation in HepG2 cells. HepG2 cell lines were seeded at $1x10^6$ in 6-well Nunc plates overnight at 37°C and 5% CO_2. They were then dosed with atabecestat (10µM-1mM) in F1 media, for 24 or 48 hours at 37°C and 5% CO_2. HepG2 cells were also dosed with 1mM glutathione for some additional conditions. Cells were washed with HBSS and then scraped, and freeze thaw was conducted twice to lyse the cells. Cells were centrifuged 14,000 rpm for 15 minutes at 4 °C. The supernatant was transferred to fresh Eppendorf tubes (1.5ml; Eppendorf, Hamburg, Germany). and cell pellets frozen. Ice-cold ACN was added to supernatant samples (following cell lysis) then centrifuged 14,000 rpm for 15 minutes at 4 °C. The samples were then dried using a Speedvac concentrator, reconstituted in 100µL LC-

MS H_20 and filtered as previously stated by using a 96-well MultiScreen filter plate (Millipore) and analysed by LC-MS/MS.

Atabecestat-GSH conjugation with or without metabolic systems. Direct conjugation of atabecestat with GSH was performed by incubation of L-glutathione reduced (GSH, 1mM, Sigma) with atabecestat (1mM) at 1:1 molar ratio at 37°C for 16hrs without metabolic system. To investigate the binding of reactive species derived from atabecestat to glutathione, GSH (200-400 µM final) was incubated with atabecestat (100-200 µM final) in the presence and absence of CYP3A4 (100 pmol/mL final, Sigma) or human liver microsomes (2 mg/mL) for two hours at 37°C. Samples were then processed using ice cold ACN to crash out proteins. Samples were then dried, reconstituted in 100 µL LC-MS H_2O, filtered for LC-MS/MS analysis.

LC-MS/MS analysis of Atabecestat-GSH conjugates. Samples derived from GSH conjugation were analysed using a Qtrap 5500 (Sciex, Framingham) coupled with an Ultimate 3000 HPLC system (Dionex, Thermo Fischer) and a C18 column (HALO C18, 50 x 2.1 mm, 2 µm, 90Å, UHPLC Column). The 16-minute gradient method consisted of 0.250 mL/min flow with increasing solvent B (5, 70 and 95% ACN). MRM transitions specific for drug-GSH adducts were selected based on mass/charge ratio (m/z) values shown in Table 1. MRM transitions were acquired at unit resolution in both the Q1 and Q3 quadrupoles to maximize specificity; they were optimized for collision energy and collision cell exit potential. MS/MS spectra were manually analysed for detection of glutathione adducts using PeakView software, version 1.5.1 (Sciex).

Table S1. MRM transitions for atabecestat-GSH conjugates.

Q1 Mass (Da)	Q3 Mass (Da)	Time (msec)	CE (volts)	ID
368.1	292.090	100.0	21.000	JNJ
543.000	292.090	100.0	28.900	JNJ-GSH
675.000	292.000	100.0	48.150	JNJ-GSH

675.000	368.000	100.0	48.150	JNJ-GSH
529.040	467.000	100.0	39.680	JNJ-GSH-N

Expression and purification of glutathione-s-transferase-Pi (GSTP). The GSTP protein was generated by transfecting a histidine tagged human GSTP gene into BL21 cells as described previously[1]. GSTP was isolated from cell lysate by binding HIS-Select Nickel Affinity Gel (Sigma-Aldrich, St Lous, MO, USA) followed by centrifugation. GSTP was characterised by SDS-PAGE using coomassie staining and then quantified using the Bradford protein quantification assay before incubation with drug.

Atabecestat-protein conjugation with or without metabolic systems. Glutathione S Transferase A1(GSTA, 100 µg, Sigma Aldrich) was reconstituted in ammonium bicarbonate buffer. Human serum albumin (HSA) (6.6 mg/ml; Sigma Aldrich, St. Louis, MO, USA) was prepared in PBS. Metabolising systems including human recombinant CYP3A4 (100 pmol/mL, Sigma Aldrich, St. Louis, MO, USA) or human liver microsomes (2 mg/mL) with β-Nicotinamide adenine dinucleotide 2′-phosphate reduced tetrasodium salt hydrate (NADPH, 1mM, Sigma Aldrich, St. Louis, MO, USA). GSTA, HSA or GSTP captured on beads were incubated with atabecestat with or without the metabolising systems for 16h. Glutathione (200 µM) was also included in selected incubations. HSA and GSTA incubations were subjected to in gel digestion, whereas GSTP incubation was processed by on-bead digestion as described previously[2]. Briefly, the beads were washed 5 times with 1000 µL phosphate buffer to remove free drug/metabolites. The protein was reduced with 10 mM dithiothreitol followed by alkylation with 50 mM iodoacetamide. The beads were washed 5 times again with 1000 µL phosphate buffer to remove excessive dithiothreitol and iodoacetamide. A suspension of beads in 30 µL of 50 mM ammonium bicarbonate buffer was incubated with 20 ng of trypsin for 16 h at 37 °C and the digests were ziptipped according to the manufacture's protocol and analysed by LC-MS/MS.

In gel digestion. Samples were prepared by addition of 4 x Laemmli sample buffer (25% glycerol (VWR Chemicals), 100 mM Tris (pH 6.8) (National Diagnostics), 2.6% SDS (Sigma-Aldrich), 1.3%

bromophenol blue (w/v) (BDH/VWR), 5% mercaptoethanol (Sigma-Aldrich)) to a final protein concentration of 1 µg/µL and then boiled at 100°C for 10 minutes. An 8% Protogel SDS-polyacrylamide gel (National Diagnostics, Atlanta, GA, USA) was made using the manufacturers protocol. 10 µg protein samples were loaded into the gel and were separated by electrophoresis (30 mA) until the length of the gel was ran. A colour metric molecular weight protein marker (Seeblue plus 2, Invitrogen, Thermo Fisher) was also loaded. Gels were fixed by 40% methanol for one hour and stained with Coomassie blue (0.08% Coomassie brilliant blue in 1.6% phosphoric acid, with 8% ammonium sulphate and 10% methanol) for two hours. The gels were then briefly destained using 10% acetic acid in 25% methanol and stored in 25% methanol. Gels were imaged using ChemiDoc Imaging systems (Bio Rad).

Gel bands were excised into 1.5mLF Eppendorf tubes and destained using 100 µL 50% ACN, 50 mM ammonium bicarbonate (ABC) solution by gentle agitation. The supernatant was removed, and bands incubated in 50 µL iodoacetamide (55mM) for 30 minutes. Supernatant was discarded and gel pieces washed three times with 50 µL 50 mM ABC. Gel pieces were rehydrated in 100 µL 10 ng/µL trypsin dissolved in 50 mM ABC and incubated overnight at 37°C. The supernatant was transferred into new lo-bind Eppendorf's. To extract peptides, the gel pieces were incubated in sonicating water bath with 30 µL 60% ACN for 5 minutes and repeated twice, pooling the collected supernatant. Supernatant was dried with a Speedvac and peptides were resuspended in 20 µL 0.1% TFA then ZipTipped for LC-MS/MS analysis.

Identification of ABCT modified proteins via mass-spectrometry. Samples were reconstituted in 2% ACN, 0.1% FA (v/v) prior to analysis using a Triple TOF 6600 mass spectrometer (Sciex) delivered into the instrument using a Eksigent NanoLC Ultra HPLC system. Samples were injected onto a nanoACQUITY UPLC Symmetry C18 Trap Column (P/N Waters, MA, USA) and washed for 10 min at 2 µL/min with 0.1% FA. A gradient from 1.6% ACN/0.1% FA to 95% ACN/0.1% FA was applied over 95 minutes at a flow rate of 300 nL/min through a Peptide BEH C18 nanoACQUITY Column (Waters, MA, USA). Spectra were acquired automatically in positive ion mode using information-dependent

acquisition powered by Analyst TF 1.5.1. software, using mass ranges of 400−1600 amu in MS and 100−1400 amu in MS/MS. Up to 25 MS/MS spectra were acquired per cycle (approximately 10 Hz) using a threshold of 100 counts per s, with dynamic exclusion for 12 s and rolling collision energy. ***Analysis of tryptic peptides.*** LC-MS/MS data were searched against the reviewed human proteome (UniProt/SwissProt accessed October 2018), using ProteinPiolot software, v5.0 (Sciex), with the following specifications: incorporating enzymatic cleavage restriction for Trypsin (maximum 3 missed cleavages, allowing for non-specific cleavage), fixed modification carbamidomethylation of cysteine, Variable modifications – methionine oxidation (+15.99), asparagine and glutamine deamidation (+0.98), ABCT modification of lysine and cysteine (+367). The maximum number of variable posttranslational modifications per peptide was 3. False discovery rate (FDR) was estimated with decoy-fusion. ABCT-modified peptides were also manually identified using characteristic fragment ions of m/z at 292.1.

GSTA modelling. Crystal structure of GSTA (PDB code 1K3L)[3] was used to generate models by removal of the ligand using Pymol (2.0, Schrodinger). GOLD 5.2 (CCDC software) [4] was used for covalent docking of ABCT to lys120, the corresponding side chain was removed from the protein and the ligand modified to contain the side chain to allow flexibility. The site of covalent attachment was at the lysine Cα. A generic algorithm with ChemPLP as the fitness function was used to generate 10 binding modes per ligand. Default settings were retained for the “ligand flexibility”, “fitness and search options”, and “GA settings.

Figure S1

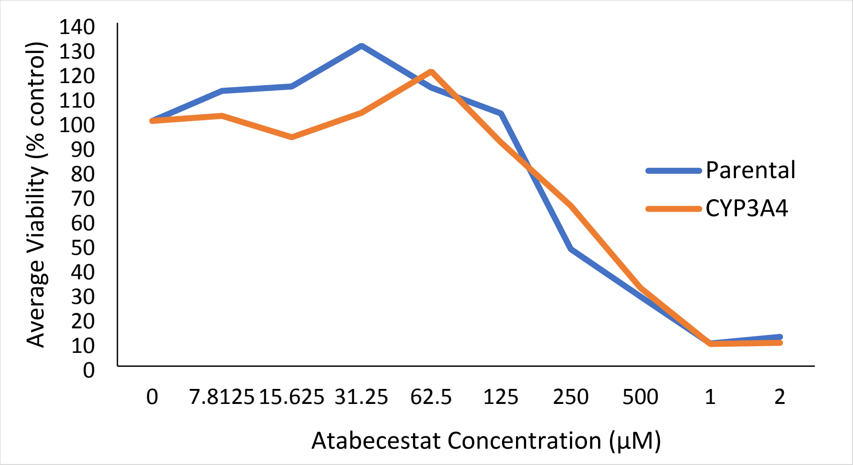

Figure S1. The toxicity of atabecestat on HepG2 cells was assessed using Cell Titer-Blue® Cell Viability Assay. HepG2 cells were incubated with 10uM-2mM atabecestat for 48hrs. Cell Titer-Blue® Cell Viability Assay (Promega) was then carried out following the manufacturer's instructions. A viability curve was then created by converting average absorbance to % viability of the control wells.

Figure S2

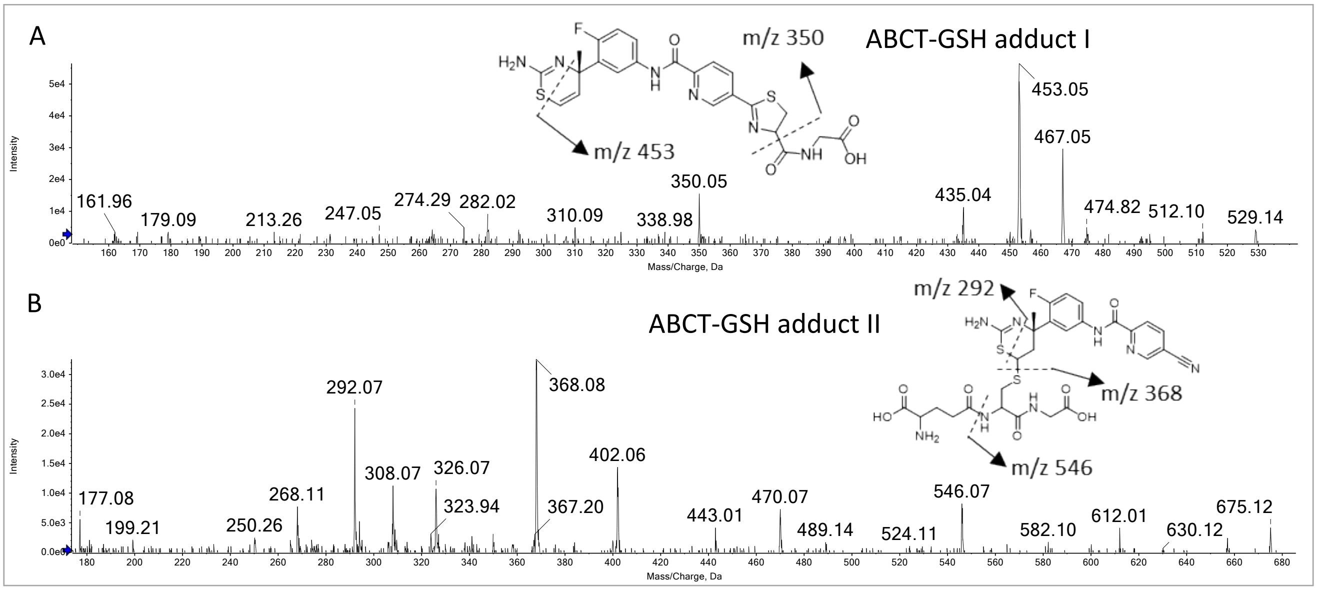

Figure S2. ABCT forms glutathione adducts with or without bioactivation. (A) ABCT-GSH adduct I with a protonated ion at m/z 529 was formed by a direct reaction between the nitrile moiety of ABCT with GSH without bioactivation; (B) in the presence of metabolic systems (CYP3A4/HepG2), ABCT was bioactivated to a reactive intermediate that reacts with GSH to form ABCT-GSH adduct II

Figure S3

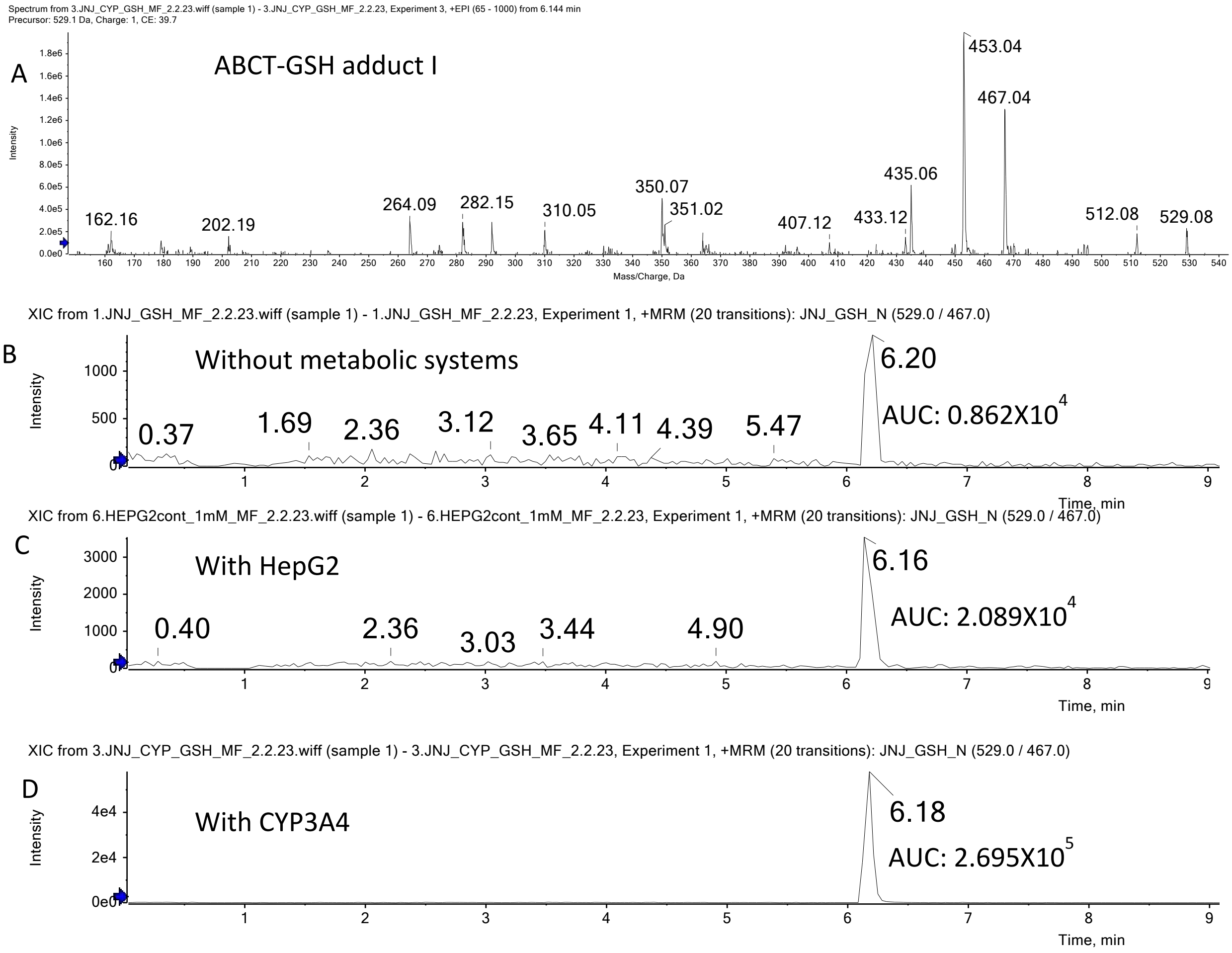

Figure S3: ABCT-GSH adduct I was formed with or without the metabolic systems. MS/MS spectrum shows the formation of ABCT-GSH adduct I in the presence of CYP3A4 (A); extracted ion chromatography of ion at m/z 529.1 corresponding to ABCT-GSH adduct I shows different levels of adduct formed without metabolic systems (B), in the presence of HepG2 cells (C) or CYP3A4 (D).

Figure S4

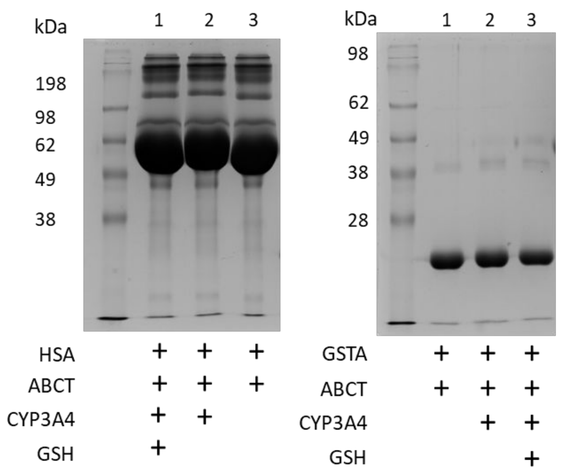

Figure S4. HSA- and GSTA-ABCT incubations were separated by SDS-PAGE. ABCT (100 µM) was incubated with HSA (A, 20 µg) and GSTA (B, 25 µg) with or without human recombinant CYP3A4 (100 pmol/mL) or glutathione (GSH, 200 µM final) for 16hr at 37 degrees. Reduced NADPH (1 mM final) was used for CYP cofactor. Proteins were separated on SDS-PAGE to remove free drug or metabolites.

Figure S5.

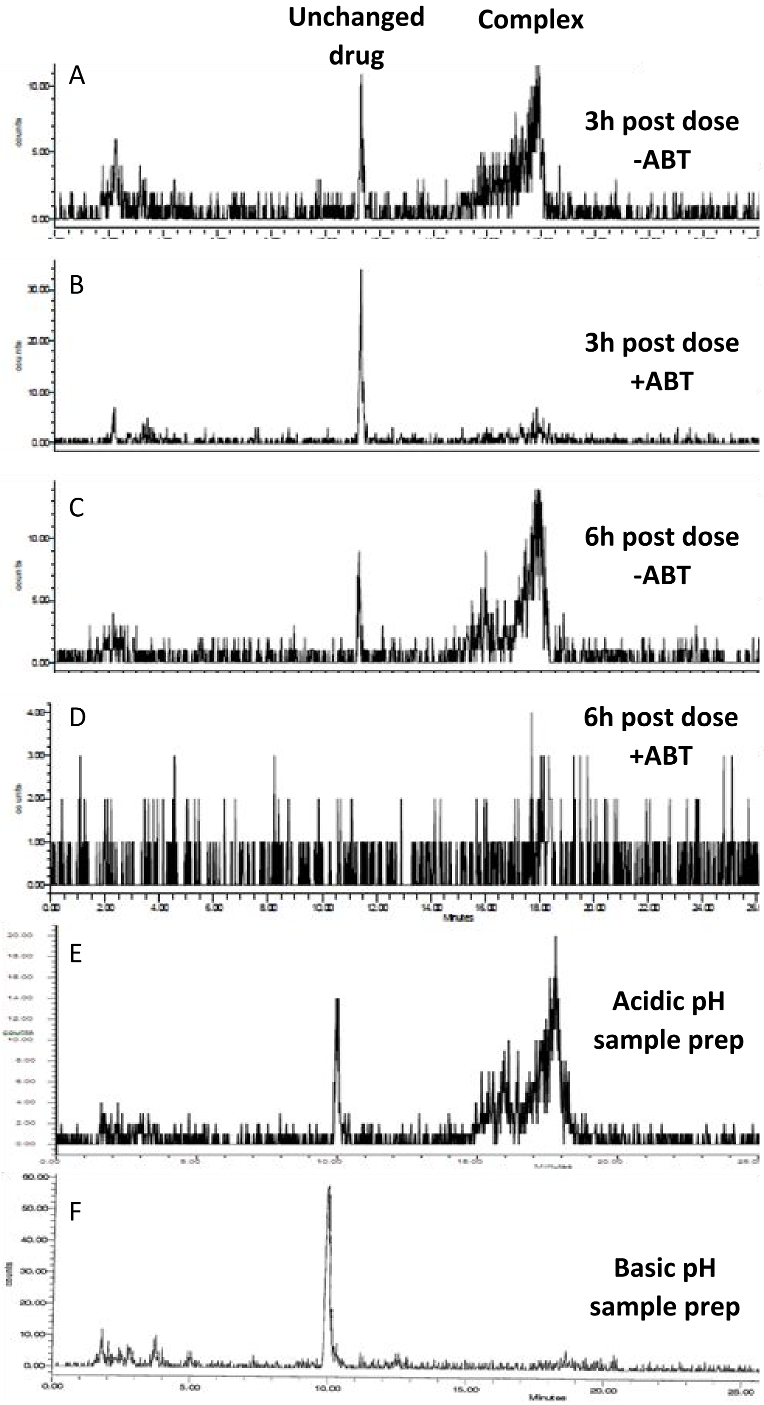

Figure S5. Radio-chromatogram of free and complex ^{14}C-atabecestat in rat plasma. Samples were collected from rats 3 (A & B) and 6h (C &D) after dosing with 3 mg/kg ^{14}C-atabecestat with or without a 100 mg/kg dose of pan-CYP inhibitor 1-ABT. Samples were prepared at acidic (E) and basic (F) conditions.

References

(1) Kitteringham, N. R., Powell, H., Jenkins, R. E., Hamlett, J., Lovatt, C., Elsby, R., Henderson, C. J., Wolf, C. R., Pennington, S. R., and Park, B. K. (2003) Protein expression profiling of glutathione S-transferase pi null mice as a strategy to identify potential markers of resistance to paracetamol-induced toxicity in the liver. *Proteomics 3*, 191.

(2) Meng, X., Waddington, J. C., Tailor, A., Lister, A., Hamlett, J., Berry, N., Park, B. K., and Sporn, M. B. (2020) CDDO-imidazolide Targets Multiple Amino Acid Residues on the Nrf2 Adaptor, Keap1. *J Med Chem 63*, 9965.

(3) Le Trong, I., Stenkamp, R. E., Ibarra, C., Atkins, W. M., and Adman, E. T. (2002) 1.3-A resolution structure of human glutathione S-transferase with S-hexyl glutathione bound reveals possible extended ligandin binding site. *Proteins 48*, 618.

(4) Naisbitt, D. J., Yang, E. L., Alhaidari, M., Berry, N. G., Lawrenson, A. S., Farrell, J., Martin, P., Strebel, K., Owen, A., Pye, M., French, N. S., Clarke, S. E., O'Neill, P. M., and Park, B. K. (2015) Towards depersonalized abacavir therapy: chemical modification eliminates HLA-B*57 : 01-restricted CD8+ T-cell activation. *AIDS 29*, 2385.
